# Clinical and pathologic characteristics of pauci-immune anti-myeloperoxidase antibody associated glomerulonephritis with nephrotic range proteinuria

**DOI:** 10.1080/0886022X.2018.1487865

**Published:** 2018-10-03

**Authors:** Peng-Cheng Xu, Tong Chen, Shan Gao, Shui-Yi Hu, Li Wei, Tie-Kun Yan

**Affiliations:** a Department of Nephrology, Tianjin Medical University General Hospital, Tianjin, China;; b Department of Hematology, Tianjin Medical University General Hospital, Tianjin, China

**Keywords:** Antineutrophil cytoplasmic antibody, nephrotic syndrome, proteinuria, histopathologic classification, pauci-immune

## Abstract

**Background:** Heavy proteinuria in antineutrophil cytoplasmic antibody (ANCA)-associated glomerulonephritis (GN) is usually considered to be associated with immune deposits in renal biopsy. Nephrotic ANCA GN without immune deposits (pauci-immune) is rare and has not been studied specially. In this study characteristics of these patients are to be investigated.

**Methods:** Clinical and pathological characteristics from 20 kidney biopsy-proven pauci-immune anti-myeloperoxidase antibody-associated GN patients with nephrotic proteinuria were analyzed and were compared with ANCA GN patients without nephrotic proteinuria.

**Results:** Acute kidney injury (AKI) and gross hematuria were much prevalent but extra-renal involvement was less prevalent in pauci-immune ANCA GN with nephrotic proteinuria than in pauci-immune ANCA GN without nephrotic proteinuria. No more severe hypoalbuminemia, hypercoagulability, hyperlipidemia or higher thrombosis incidence were found between two groups. Compared with patients without nephrotic proteinuria, patients with nephrotic proteinuria had more prevalent crescentic category in histopathology. Proteinuria decreased quickly after treatment but much poorer renal prognosis was found in pauci-immune ANCA GN with nephrotic proteinuria. The results of urinary albumin to total protein ratio and urinary protein electrophoresis showed pauci-immune ANCA GN with nephrotic proteinuria had obvious non-selective proteinuria.

**Conclusions:** Pauci-immune ANCA GN with nephrotic proteinuria do not have more severe hypoalbuminemia, hypercoagulability or hyperlipidemia than patients without nephrotic proteinuria. Non-selective proteinuria might be the reason. However, pauci-immune ANCA GN with nephrotic proteinuria have more prevalent crescentic category in histopathology, higher incidence of AKI, gross hematuria and poorer renal prognosis despite of good sensitivity to therapy of proteinuria.

## Introduction

Antineutrophil cytoplasmic antibody (ANCA)-associated vasculitis (AAV) is characterized by positive serum ANCA and has a high proportion of kidney injury with pauci-immune necrotizing glomerulonephritis (GN) as the most common renal histopathological manifestation [1–5]. Nephrotic syndrome includes a series of diseases which is characterized by heavy proteinuria (>3.5 g per 1.73 m^2^ body surface area per day) and often associated with hypoalbuminaemia, hypercoagulability and hyperlipidemia [6], but these characteristics are not invariable in some proliferative GN [7]. ANCA GN with nephrotic proteinuria is rare and the clinical and pathological characteristics of these patients have not been elucidated by now. The sensitivity to therapy of heavy proteinuria varies greatly in different kinds of nephrotic syndrome, which influence the prognosis of kidney. So the renal prognosis of ANCA GN with nephrotic proteinuria needs to be studied. On the other hand, the existence of hypercoagulability in most patients with AAV has been demonstrated [8]. Since hypercoagulability is an important feature of nephrotic syndrome, whether AAV with nephrotic proteinuria has more severe hypercoagulability also needs to be investigated.

In this study, we collected 20 kidney biopsy-proven AAV patients with pauci-immune GN and nephrotic proteinuria and compared them with 112 patients without nephrotic proteinuria, in order to explore the intrinsic characteristics of AAV with nephrotic proteinuria. Previous studies suggested that heavy proteinuria in patients with ANCA GN was associated with glomerular immune deposits [9–11]. ANCA GN has also been reported to occur superimposed on other glomerular disease processes which are characterized by glomerular immune deposits [12–17]. To exclude the interference of the potential coexisting other types of GN, only patients with pauci-immune ANCA GN were enrolled in this study.

## Materials and methods

### Participants

We included 148 patients with Myeloperoxidase (MPO)-ANCA associated vasculitis with renal biopsies between January 2005 and October 2017 in the Tianjin Medical University General Hospital. All the patients fulfilled the Chapel Hill Consensus Conference classification [18] and were routinely tested for ANCA and antiglomerular basement membrane antibody. Renal biopsies were performed for patients at the time of diagnosis. Renal specimens were evaluated using direct immunofluorescence and light microscopy. Intensity of staining was scored as negative (–), mild (+), moderate (++) or strong (+++). Biopsy without immune deposits (pauci-immunity) was defined as no more than 2+ intensity of immunostaining and no detectable electron-dense deposits on electron microscopy [19]. Exclusion criteria were proteinase 3 (PR3)-ANCA positivity, immune deposits in biopsy, diabetes, hepatitis, cirrhosis, pregnancy, previous malignancy, infection at onset of the disease and positive antiglomerular basement membrane antibody. Sixteen patients were excluded because of immune deposits in biopsy. The other 132 patients (20 with nephrotic proteinuria and 112 without) were included in our study at last. The research was in compliance of the declaration of Helsinki and the protocol was approved by the institutional review board of Tianjin Medical University General Hospital (IRB2013–001-01). Informed consent was obtained from all individual participants included in the study.

### Clinical and laboratory findings

Clinical data included the following: gender, age (years), time from onset (days) and the level of Birmingham Vasculitis Activity Score (BVAS). Acute kidney injury (AKI) was defined by Kidney Disease Improving Global Outcomes (KDIGO) in 2012 [20]. Fever was defined as body temperature >38.5 °C. Weight loss was defined as loss of >2 kg over 1 months preceding diagnosis. Extra-renal manifestations included following items. Skin involvement was defined as new skin lesions during the active period of AAV which could not be explained by any other cause. Mucous/Eye involvement included oral/genital ulcer, conjunctivitis, uveitis and fundus hemorrhage. Ear involvement included hearing loss and otitis media. Nasal involvement included sinusitis, nasal polyposis and epistaxis. Lung involvement was diagnosed when there was hemoptysis or chest x-ray and/or computed tomography revealed nodule or infiltration. Digestive tract involvement was diagnosed based on the existence of gastrointestinal bleeding or the positive result of fecal occult blood. Peripheral neuropathy was diagnosed if new mononeuropathy or multiple mononeuropathies occurred. Neuropathy was diagnosed once other causes of neuropathy were excluded.

Laboratory data included the following: blood routine test, albumin, proteinuria, serum creatinine (Scr), cholesterol, triglycerides, erythrocyte sedimentation rate (ESR), C reactive protein (CRP), Complement 3 (C3) and 4 (C4). Proteinuria was defined as urine total protein is more than upper normal limit (>0.15 g/24 h). Nephrotic proteinuria was defined as urine total protein ≥3.5 g/24 h [21]. Estimated glomerular filtration rate (eGFR) was calculated with an equation developed by adaptation of the modification of diet in renal disease equation on the basis of data from Chinese chronic kidney disease patients [22].

### Renal histopathology

Renal histopathology was evaluated by two pathologists who were blinded to clinical data of patients. Each glomerulus was scored on the presence of fibrinoid necrosis, crescents (cellular/fibrous) and global glomerulosclerosis. The presence of glomerular lesions was calculated as the percentage of the total number of glomeruli in a biopsy. The pathological classification was described previously in [5].

### Urinary protein electrophoresis

Morning urine 10 mL was collected and centrifugated at 1500 rpm for 5 min. The protein concentration was adjusted to be 2+ with normal saline. Urine was combined with Sodium Dodecyl Sulfonate-bromophenol blue diluent as a ratio of 4:1 and was loaded with a volume of 5 μL (Hydrasys sebia France). The sample was electrophoresed for 15 min. Then the gel was dried, dyed, decolorized fixed and scanned with electrophoresis gel imaging system (Syngene GeneGenius). The urinary protein was defined as high-molecular weight protein (HMW, relative molecular mass >70 kD), middle-molecular weight protein (MMW, relative molecular mass =70 kD) and low-molecular weight protein (LMW, relative molecular mass <70 kD) according to the position of protein in gel.

### Statistical analyses

Differences of quantitative parameters between groups were assessed using the *t* test (Independent two-sample *t*-test or paired two-sample *t*-test, for data that were normally distributed) or nonparametric test (Mann-Whitney *U* test, for data that were not normally distributed). Categorical variables are presented as frequencies. The mortality rate was calculated with the Kaplan-Meier method and the curves were compared using the Log-Rank test. The *p* values < .05 were considered significant. The software SPSS, version 19.0 for Windows (IBM, Chicago, IL, USA), was used for statistical analysis.

## Results

### Comparison of the clinical and laboratory characteristics between ANCA GN with and without nephrotic proteinuria

As shown in Table 1, no difference of the gender, age, time from onset and BVAS was found between patients with and without nephrotic proteinuria. The kidney injury of patients with nephrotic proteinuria was more severe. The incidence of AKI, dialysis on admission, gross hematuria and edema in patients with nephrotic proteinuria were higher than that in patients without nephrotic proteinuria (*p* = .016, .019, .019 and .020 respectively). There was a reverse trend as for the extra-renal manifestations. The incidence of weight loss, involvement of ear/nose/throat and involvement of nervous system in patients with nephrotic proteinuria were lower than that in patients without nephrotic proteinuria (*p* = .011, .046 and .040 respectively). No significant difference of other organ involvement was found.

**Table 1.** Comparison of clinical features of patients with and without nephrotic proteinuria.

Feature	Non-nephrotic proteinuria (*n* = 112)	Nephrotic proteinuria (*n* = 20)	*p* value
Male/female	63/49	9/11	.352
Age (years)	59.00 ± 14.18	54.55 ± 17.52	.215
Time from onset (days)	90 (14, 210)	90 (14, 150)	.625
BVAS	20.27 ± 5.38	20.15 ± 5.80	.924
Acute kidney failure (Y/N)	86/26	20/0	.016
Dialysis on admission (Y/N)	19/93	8/12	.019
Gross hematuria (Y/N)	23/89	9/11	.019
Edema (Y/N)	32/80	11/9	.020
Fever (Y/N)	62/50	13/7	.423
Weight loss (Y/N)	51/61	3/17	.011
Muscle/joint (Y/N)	38/74	3/17	.092
Skin (Y/N)	16/96	4/16	.511
Eyes/mucous membranes (Y/N)	20/92	2/18	.385
Ear/nose/throat (Y/N)	49/63	4/16	.046
Lung (Y/N)	50/62	10/10	.658
Cardiovascular (Y/N)	5/107	0/20	.335
Digestive tract (Y/N)	16/96	0/20	.071
Nervous system (Y/N)	20/92	0/20	.040

BVAS: Birmingham vasculitis activity score.

Laboratory characteristics between ANCA GN with nephrotic proteinuria and ANCA GN without nephrotic proteinuria was compared in Table 2. The initial eGFR and hematuria level of patients with nephrotic proteinuria was significantly lower than that of patients without nephrotic proteinuria (*p* < .001 and *p* = .018, respectively). No significant differences of the other laboratory characteristics including D-Dimer, serum albumin cholesterol and triglycerides were found between two groups.

**Table 2.** Comparison of laboratory features of patients with and without nephrotic proteinuria.

Feature	Non-nephrotic proteinuria (*n* = 112)	Nephrotic proteinuria (*n* = 20)	*p* value
ANCA level (IU/mL)	98.95 ± 39.41	114.63 ± 53.47	.124
eGFR (mL/min/1.73m^2^)	36.05 ± 35.62	12.33 ± 9.24	<.001
Proteinuria (g/24h)	1.35 ± 1.00	4.61 ± 1.13	<.001
Hematuria (/μL)	226.49 ± 187.36	503.47 ± 422.49	.018
Hemoglobumin (g/L)	8.98 ± 2.16	8.55 ± 1.74	.403
White blood cell (10^9^/L)	9.83 ± 3.37	10.34 ± 6.95	.751
Platelet (10^9^/L)	30.21 ± 14.74	28.81 ± 11.45	.687
D-Dimer (ng/mL)	2628.94 ± 2044.55	2857.65 ± 2224.28	.650
ESR (mm/h)	83.54 ± 40.13	86.10 ± 35.38	.789
Serum albumin (g/L)	31.42 ± 3.77	30.05 ± 3.16	.129
Cholesterol (mmol/L)	4.12 ± 1.06	4.28 ± 1.11	.562
Triglycerides (mmol/L)	1.34 ± 0.59	1.45 ± 0.59	.336
Complement 3 (mg/dL)	93.41 ± 21.61	94.70 ± 16.83	.801
Complement 4 (mg/dL)	36.35 ± 17.63	31.95 ± 12.81	.289
C reactive protein (mg/dL)	3.39 (0.13, 23.90)	5.07 (0.84, 22.80)	.088

ANCA: antineutrophil cytoplasmic antibody; eGFR: estimated glomerular filtration rate; ESR: erythrocyte sedimentation rate.

### Comparison of the renal histopathological characteristics between ANCA GN with and without nephrotic proteinuria

**Figure 1.**
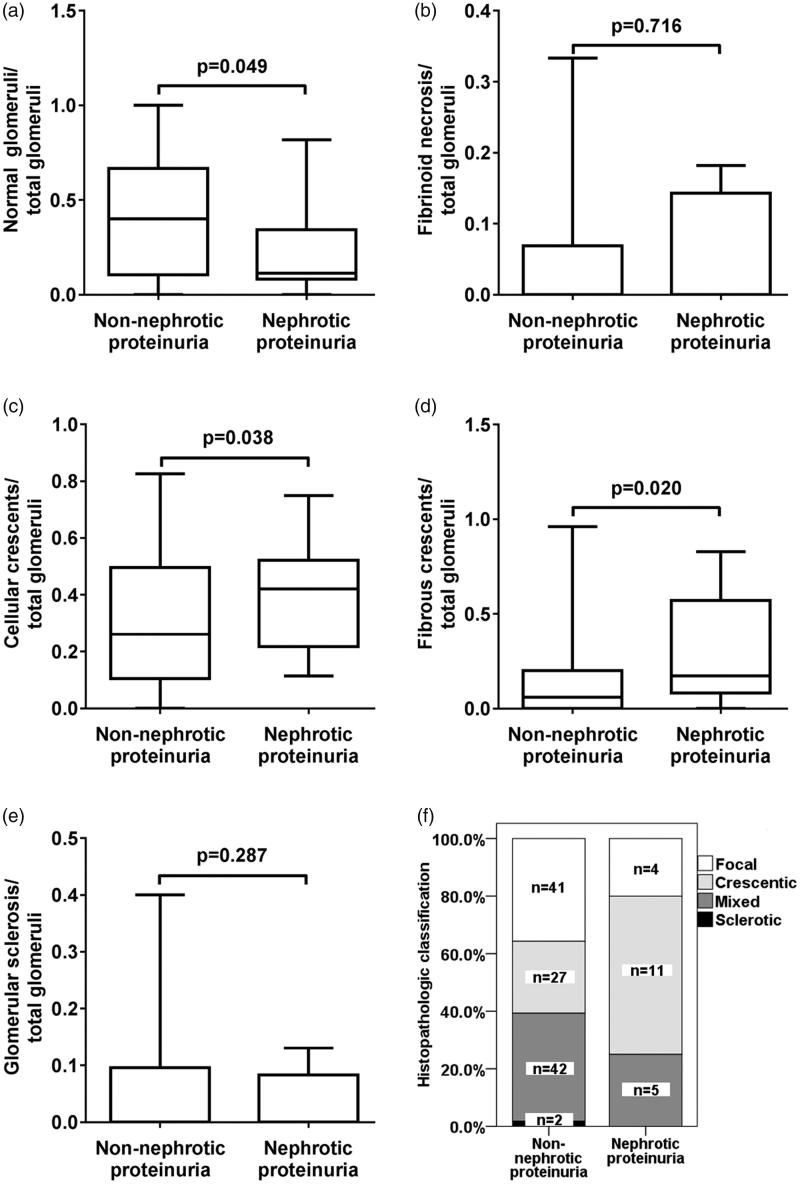
Comparison of the glomerular lesions between ANCA GN with and without nephrotic proteinuria. (A) Comparison of the ratio of normal glomeruli to total glomeruli between ANCA GN with and without nephrotic proteinuria (B) Comparison of the ratio of fibrinoid necrosis to total glomeruli between ANCA GN with and without nephrotic proteinuria. (C) Comparison of the ratio of cellular crescents to total glomeruli between ANCA GN with and without nephrotic proteinuria. (D) Comparison of the ratio of fibrous crescents to total glomeruli between ANCA GN with and without nephrotic proteinuria. (E) Comparison of the ratio of glomerular sclerosis to total glomeruli between ANCA GN with and without nephrotic proteinuria. (F) Comparison of the histopathologic classification between ANCA GN with and without nephrotic proteinuria.

The percentage of normal glomeruli in total glomeruli of patients with nephrotic proteinuria was lower than that of patients without nephrotic proteinuria (*p* = .049). The percentage of cellular and fibrous crescents in total glomeruli of patients with nephrotic proteinuria were higher than that of patients without nephrotic proteinuria (*p* = .038 and .020, respectively). No differences of the fibrinoid necrosis and glomerular sclerosis were found between two groups (Figure 1(A–E)).

The renal pathology was classified according to the histopathological classification [5] and compared between two groups (Figure 1(F)). Of the 20 patients with nephrotic proteinuria, 4, 11, 5 and 0 patients were classified as focal, crescentic, mixed and sclerotic type respectively. Of the 112 patients without nephrotic proteinuria, 41, 27, 42 and 2 patients were classified as focal, crescentic, mixed and sclerotic type, respectively. Crescentic type was more prevalent in patients with nephrotic proteinuria than that in patients without nephrotic proteinuria (*p* = .044).

### Comparison of the prognosis between ANCA GN with and without nephrotic proteinuria

**Figure 2.**
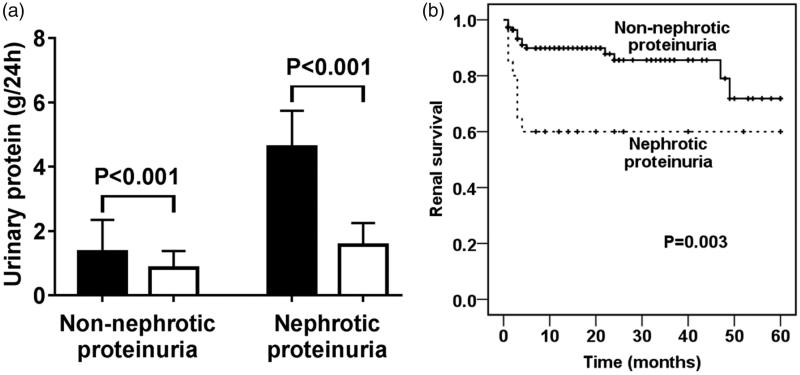
Comparison of the prognosis between ANCA GN patients with and without nephrotic proteinuria. (A) Comparison of the proteinuria before and after treatment of patients with and without nephrotic proteinuria. Dark column: before treatment. Hollow column: after treatment. (B) Comparison of the mortality between patients with and without nephrotic proteinuria.

All patients received corticosteroid treatment. The majority of patients (126 of 132) also received intravenous or oral cyclophosphamide. Treatment protocols were comparable between patients with and without nephrotic proteinuria. The proteinuria of all patients with nephrotic proteinuria decreased to be <3.5 g/24 h within 1 months after the initiation of treatment (Figure 2(A)). Among eight patients who had nephrotic proteinuria and needed hemodialysis on admission, two patients got rid of hemodialysis within 1 month after the initiation of treatment and the other six patients developed end stage renal disease (ESRD) finally. Two patient who did not need hemodialysis on admission developed ESRD during follow-up. When compared with the patients without nephrotic proteinuria, patients with nephrotic proteinuria had poorer renal survival (*p* = .003) (Figure 2(B)).

### Urinary protein electrophoresis of ANCA GN with nephrotic proteinuria

The urine of 12 patients with ANCA GN with nephrotic proteinuria was collected for urinary protein electrophoresis. The percentage of MMW proteinuria (representing albuminuria) in total proteinuria of ANCA GN with nephrotic proteinuria was 71.25 ± 5.97. The percentage of LMW proteinuria in total proteinuria of ANCA GN with nephrotic proteinuria was 12.33 ± 4.92. The percentage of HMW proteinuria in total proteinuria of ANCA GN with nephrotic proteinuria was 16.42 ± 8.38.

## Discussion

No consistent conclusion about the relationship between proteinuria and renal prognosis of AAV has been drawn by previous studies [23–25]. Hauer et al scored the clinical and histological features of 96 patients with renal biopsies and found the proteinuria did not predict the eGFR at 18 months [24], but in that study the patients with immune deposits in renal biopsies were not excluded. Yorioka et al. [25] found that patients with MPO-ANCA GN and did not survive during follow-up had a lower serum albumin and creatinine clearance than those who survived, but there was no difference of urinary protein between two groups. However, only 17 cases were enrolled in this study. Pauci-immune necrotizing crescentic GN is the most common histopathological type of ANCA GN [3–5] and nephrotic range proteinuria is relatively rare. It is worth noting that ANCA GN, especially those with high levels of proteinuria, have been reported to often occur superimposed on other glomerular disease processes which are characterized by glomerular immune deposits [9–17]. In these cases, proteinuria may be potentiated by synergetic effects of the coexisting two types of diseases. To exclude the interference of the potential coexisting other types of GN, only patients with pauci-immune GN were enrolled in this study. We found pauci-immune ANCA GN with nephrotic proteinuria had severe lesions in renal pathology and a high incidence of ESRD.

Although massive proteinuria is the initiating disorder in NS, the precise pathogenesis of hypoalbuminemia in NS has not been fully elucidated, because some patients with nephrotic proteinuria do not have hypoalbuminemia [7]. In this study, we found that there was no difference of serum albumin between ANCA GN patients with and without nephrotic proteinuria. This phenomenon indicates that hypoalbuminemia might not, at least not totally be induced by proteinuria in AAV. Some possible explanations could be speculated. First, ANCA GN is a kind of severe proliferative nephritis. The coexisting tubulointerstitial damage caused non-selective proteinuria. Second, hypoalbuminemia not only can be the consequence of massive proteinuria, but also can be the result of the combined effects of inflammation and inadequate nutrition intake in patients with inflammatory disease. Third, capillary permeability might be an important factor influencing the emerging of hypoalbuminaemia in nephrotic syndrome [26].

Besides massive proteinuria, NS is often characterized by hypoalbuminaemia, hypercoagulability and hyperlipidemia [6]. We found ANCA GN with nephrotic proteinuria was not accompanied by hyperlipidemia, but hypercoagulability (expressed by high levels of D-Dimer) was very prominent in ANCA GN with nephrotic proteinuria. However, we did not think the hypercoagulability in ANCA GN with nephrotic proteinuria is a manifestation of nephrotic syndrome because ANCA GN without nephrotic proteinuria also had a comparable level of D-Dimer. Actually, it had been observed in a number of studies that there is high prevalence of venous thromboembolism in patients with AAV [27]. The levels of circulating D-Dimer are significantly higher in AAV patients independent of proteinuria [8]. Thus, ANCA GN with nephrotic proteinuria is not a typical NS.

Renal involvement is considered as a severe disease manifestation of AAV. Although immunosuppressive therapy may be lifesaving, many patients still progress to ESRD. The histopathological classification proposed by Berden et al. in 2010 is the best classification system to predict the renal outcomes. In the current study we found crescentic type was more prevalent in ANCA GN with nephrotic proteinuria than that in ANCA GN without nephrotic proteinuria. This phenomenon corresponded with the poor renal prognosis in ANCA GN with nephrotic proteinuria even though the proteinuria was sensitive to treatment. An interesting phenomenon needs further study was the relatively less extra-renal involvements in ANCA GN with nephrotic proteinuria.

Several limitations of this study should be mentioned. Since the pauci-immune ANCA GN with nephrotic proteinuria is not common, the small sample size in this study might influence the accuracy of the results, so further study including large sample is needed. Since the AAV with positive PR3-ANCA is rare in Chinese and only MPO-ANCA positive patients were included, whether the result of the current study is also suitable for patients with positive PR3-ANCA is unknown.

## Conclusions

In conclusion, ANCA GN with nephrotic proteinuria has non-selective proteinuria. Compared with ANCA GN without nephrotic proteinuria, ANCA GN with nephrotic proteinuria do not have more severe hypoalbuminemia, hypercoagulability and hyperlipidemia, but has less extra-renal involvements, more severe renal involvement and higher percentage of crescentic type in renal pathology. The incidence of ESRD is also higher in ANCA GN with nephrotic proteinuria.
